# “You sure she's not making this up?”: A qualitative investigation of stigma toward adults with borderline personality disorder in physical healthcare settings

**DOI:** 10.1002/pmh.1646

**Published:** 2024-11-24

**Authors:** Kellyann M. Navarre

**Affiliations:** ^1^ Cleveland State University Cleveland OH USA

## Abstract

Borderline personality disorder (BPD) is associated with pervasive stigma that contributes to several consequences, such as inaccessible and inadequate healthcare. Existing literature concerning the experiences of BPD stigma within healthcare settings predominantly centers on mental healthcare contexts. However, individuals with BPD also present with elevated physical disabilities and health concerns, demonstrating a need for regular contact with medical professionals to manage and coordinate physical healthcare. The current qualitative study analyzes the dynamics of stigma and consequences in medical settings for physical healthcare among individuals diagnosed with BPD. Community adults (*N* = 16, *M*_*age*_ = 29.50, 44% cisgender women) provided qualitative responses describing their experiences with stigma in medical settings other than for mental health purposes. The inductive thematic analysis generated six key themes: (1) Dismissal and Misattribution of Physical Symptoms, (2) Delayed or Inappropriate Medical Diagnosis and Intervention, (3) Communication and Advocacy Challenges, (4) Invalidation of Emotional Well‐Being and Distress, (5) Self‐Harm Stigma, and (6) Presumed Drug‐Seeking Behavior. This article demonstrates the persistent and complex role of stigma across physical healthcare settings for individuals with BPD, affecting their physical and mental healthcare outcomes. It also identifies areas for future research and improvement and offers insights to ameliorate these issues.

## INTRODUCTION

While stigma affects all psychological disorders, the stigma associated with borderline personality disorder (BPD) is exceptionally pervasive and challenging. Research suggests that BPD may be more stigmatized than other highly stigmatized psychological disorders, including other personality disorders and schizophrenia (Markham, 2003; Markham & Trower, 2003; Masland & Null, 2022; Sheehan et al., 2016). Clinicians may misconstrue individuals with BPD as having more control over their behaviors compared to people with other psychological disorders, thus perceiving their difficulties as a personal failing or deliberate misbehavior (Sheehan et al., 2016; Sulzer, 2015). BPD encompasses experiences of intense and dysregulated emotions, frequently triggered within interpersonal interactions, alongside high rates of recurring suicidality and self‐injury to downregulate distress (American Psychiatric Association [APA], 2022; Black et al., 2004; Goodman et al., 2017; Lazarus et al., 2014). Interpersonal difficulties, such as extreme reactions to abandonment or rejection, render BPD particularly prone to stigma (Aguirre, 2016; Aviram et al., 2006; Kealy & Ogrodniczuk, 2010; Sansone & Sansone, 2013). Furthermore, the affective instability that is prone to intensify and change quickly, intertwined with challenges maintaining a stable self‐concept and self‐destructive impulsivity, are often associated with derogatory labels like “manipulative” or “attention‐seeking,” rather than recognized as genuine signs of distress and a need for support (Agnol et al., 2019; Aguirre, 2016; Aviram et al., 2006; Masland et al., 2022; Ociskova et al., 2017). The literature consistently highlights a pattern in which the difficulties of individuals with BPD are overlooked, and the recounting of their experiences is perceived as lacking credibility.

BPD stigma impedes access to adequate mental healthcare and exacerbates preexisting difficulties of those living with the disorder (e.g., Ociskova et al., 2017). This stigma is evident in several ways, including the dissemination of stigmatizing information, poor mental health literacy about BPD, decreased treatment‐seeking behavior, and hesitation to give a diagnosis (Aviram et al., 2006; Klein et al., 2022; Lohman et al., 2017; Wall et al., 2021). Mental health professionals have reported increased negative attributions, reduced empathy, and unhelpful adjustments in their behavior and treatment approach, such as avoidance, when working with individuals with BPD (Aviram et al., 2006; Forsyth, 2007; Ociskova et al., 2017). Although the stigma may be especially present in professional spheres, namely research and clinical settings, BPD stigma persists in the general public as well (Bowen, 2019; Elliott & Ragsdale, 2024; Masland & Null, 2022). Moreover, there is some limited evidence indicating that BPD stigma extends to medical settings for physical healthcare. For instance, chronic pain professionals have described BPD patients, when making treatment management recommendations, as individuals who falsify information and distort their experiences (Saper & Lake, 2002). There is also limited qualitative research on stigma in medical care settings toward mental health and substance use disorders more broadly (Cunningham et al., 2023; Zirnsak et al., 2024). Among these studies, regarding BPD, one reported a participant whose health was incorrectly attributed to their BPD diagnosis (Zirnsak et al., 2024); however, studies have not explored BPD stigma in medical settings.

Stigmatizing generalizations in healthcare settings are particularly concerning in light of evidence revealing the intersection between BPD and co‐occurring physical health conditions and complex medical needs (e.g., El‐Gabalawy et al., 2010). A variety of mechanisms for the relationship between BPD and physical health issues have been examined, including emotion regulation difficulties and physiological responses to chronic stress (Cohen et al., 2012; Gratz et al., 2017; Sapolsky et al., 2000). The presence of BPD or high BPD symptomatology is associated with higher rates of arthritis, heart disease, diabetes, and other medical conditions (Barber et al., 2020; El‐Gabalawy et al., 2010), as well as chronic pain (Braden & Sullivan, 2008; McWilliams & Higgins, 2013; Reynolds & Tragesser, 2018). Nonremitted BPD, compared to remitted occurrences, is associated with an increased risk for chronic physical health concerns (e.g., back pain, fibromyalgia; Frankenburg & Zanarini, 2004). In one study, 60% of patients with BPD received Social Security Disability Income at some point in their lives (Zanarini et al., 2009). Additionally, BPD symptoms are a prospective predictor of physical health symptoms, such as headaches, dizziness, colds, and coughs (Gratz et al., 2017). Given these elevated rates of physical disabilities and health concerns, individuals with BPD may be in considerable need of ample medical care and support.

Addressing stigma and inadequate care in these settings is especially essential given the elevated risk of suicide among individuals with BPD who have co‐occurring physical health conditions, as well as the increased need to use costly medical treatment (El‐Gabalawy et al., 2010; Frankenburg & Zanarini, 2004). As a result of their medical needs, individuals with BPD have a greater need for frequent primary care utilization, requiring ongoing contact with medical professionals (Dubovsky & Kiefer, 2014; Gross et al., 2002). However, the prevailing discourse on BPD stigma tends to concentrate on mental healthcare contexts, overlooking the potential stigma present in other healthcare domains. There remains a dearth of research on how this stigma may contribute to physical health disparities in medical settings among adults diagnosed with BPD when they seek care. The stigma attached to BPD warrants inquiry within other relevant healthcare settings that have yet to be explored to offer insights for identifying and rectifying these issues. Traditionally, professionals have been regarded as the primary experts in defining and describing patients' experiences, with limited research signifying social narratives to understand stigmatized communities (Moller et al., 2021; Schleider, 2023). Thus, the purpose of this study is to examine the manifestation of stigma from the perspective of individuals diagnosed with BPD, particularly in relation to their intersecting physical disabilities and physical health concerns. By qualitatively exploring the stigma within medical healthcare settings, the study aims to identify barriers that impede access to quality healthcare and understand their corresponding consequences. Additionally, the study aims to amplify the perspectives of individuals directly affected and promote an informed approach to healthcare delivery that is responsive to the needs of individuals with BPD.

## METHOD

### Procedure

Participants in the current qualitative study (*N* = 16) were recruited within a larger study (*N* = 81) about lived experiences of BPD. Adults from the community who identified as being diagnosed with borderline personality disorder responded to an online survey administered on a secured server. All participants were recruited through reputable referrals and sources, including national nonprofit organizations and community support groups managed by peer workers and professionals. Inclusion criteria for the study required participants to be adults diagnosed with BPD, aged 18 or older, proficient in English, and residing in either the United States or Canada. Individuals who expressed interest in the study via telephone or email were contacted to participate in an online survey that lasted approximately 45 minutes. The lead author had prior acquaintance with some of the individuals who expressed interest due to the community‐driven nature of the study (Collins et al., 2018). Participant information was not associated with study data to maintain confidentiality. Each participant was reminded about confidentiality and provided informed consent, including permission for their responses to be used for research purposes. Participants were informed that the study, conducted by the lead author, aimed to better understand and advocate for the lived experiences of individuals with BPD, including stigma. They were required to complete a comprehension check before beginning the study, and all survey items were screened for consistency and validity. The study also obtained approval to ask several screening questions to capture consistency and information about participants' diagnosis, detailing the diagnosing professional and timing of diagnosis, as well as various measures of BPD (see Table 1).

**TABLE 1:** Demographic breakdown of participants by sample size.

Characteristic	Response	*N* (%)		
		*N* = 81	*N* = 29	*N* = 16
1. Age (*mean/standard deviation*)		31.93 (11)	30.93 (8)	29.50 (5)
2. PAI‐BOR (*mean/standard deviation*)		50.07 (11)	49.00 (10)	49.9 (9)
3. Sex	Female	55 (68)	18 (62)	11 (69)
	Male	26 (32)	11 (38)	5 (31)
4. Race/ethnicity	^a^White/European	38 (47)	16 (55)	8 (50)
	^b^White/European	56 (69)	19 (66)	12 (75)
	Black/African American	17 (21)	8 (28)	5 (31)
	Hispanic/Latino/a/x	16 (20)	6 (21)	2 (13)
	Native American	5 (6)	3 (10)	2 (13)
	Middle eastern/north African	1 (1)	‐	‐
	Pacific islander	1 (1)	1 (3)	‐
	South Asian	2 (2.5)	‐	‐
	Southeast Asian	1 (1)	‐	‐
	East Asian	3 (4)	‐	‐
5. Gender	Cisgender women	40 (49)	12 (41)	7 (44)
	Cisgender men	24 (30)	10 (35)	5 (31)
	Gender minority	17 (21)	7 (24)	4 (25)
6. Comorbidity	Psychological disorder	65 (80)	21 (72)	13 (81)
	Physical disability/chronic illness	44 (54)	21 (72)	13 (81)

*Note*: Participants were permitted to select more than one race/ethnicity response option. PAI‐BOR = Personality Assessment Inventory‐Borderline Scale.

^a^ White/European = Participants who selected only White/European.

^b^ White/European = Participants who selected White/European along with another racial/ethnic identity (multiracial).

### Materials

Participants responded to an online qualitative survey designed for the purposes of the study. The use of online qualitative surveys offers several advantages and is considered equally suitable for qualitative research compared to other approaches, such as interviews (Braun et al., 2020). Participants were initially queried with a dichotomous question (Yes/No) that asked: Have you ever experienced stigma related to your BPD diagnosis or BPD symptoms in a medical setting other than for mental health purposes (e.g., when receiving medical care for wounds, injuries, accidents, medical conditions, etc.)? This approach aimed to understand the frequency and potential impact of these experiences on quality of care and patient outcomes. Subsequently, participants who indicated having encountered such stigma were presented with the optional opportunity to expand upon their experiences through an open‐ended prompt to provide personalized, in‐depth qualitative descriptions. This prompt instructed participants to elaborate about their experiences using written responses. The online survey tool provided an open‐ended question structure, with no leading questions.

### Participants

In total, *N* = 16 participants provided qualitative responses to the current study question. This subset includes individuals who, after endorsing the earlier question about BPD stigma in a medical setting, chose to disclose further and provide optional qualitative responses to the supplemental study question. This sample size is sufficient and aligns with qualitative research practices, following the reflexive thematic analysis standards of “information power.” This framework considers the inductive and reflexive study aims, analysis strategy, participant specificity, existing research and frameworks related to BPD stigma, and the quality of the responses that generated consistent themes (Braun & Clarke, 2022; Braun & Clarke, 2024; Malterud et al., 2016). Participant ages ranged from 22 to 40, with a mean age of 29.50 years (*SD* = 5.38). While 69% of participants reported being assigned female at birth (*N* = 11) and 31% of participants reported being assigned male at birth (*N* = 5), 44% of participants identified as cisgender women (*N* = 7), and 25% of participants identified as nonbinary, transgender, genderqueer, and/or genderfluid (*N* = 4). The full participant demographic characteristics, including from the larger study, are reported in Table 1.

### Qualitative data analysis

A critical realist reflexive thematic analysis of the written responses was conducted by systematically examining the dataset (Braun & Clarke, 2022; Byrne, 2021). This study was informed by applicable reporting standards outlined in the Consolidated Criteria for Reporting Qualitative Research (COREQ; Tong et al., 2007). Taking into account the limitations of these standards for the qualitative approach in this study, the Reflexive Thematic Analysis Reporting Guidelines (RTARG) were also adopted (Braun & Clarke, 2024). Given the nature of the research question and the limited existing literature on this topic, a primarily inductive, data‐driven approach was used to develop the codes and construct themes within the data. Inductive analysis, compared to deductive analysis, involves generating codes and themes from the data, rather than starting with codes prior to analysis. This approach is useful for highlighting complex lived experiences and perspectives, particularly in research areas with limited existing research (Braun & Clarke, 2006; Moller et al., 2021). The flexible applicability of a reflexive approach enables analyses to be informed by existing research on stigma, lived experiences, and community‐based research, while also situating marginalized experiences within their contextual and social meanings (Braun & Clarke, 2022). This methodological engagement illuminates the experiences of socially marginalized communities and allows for reconsideration of dominant discourses that perpetuate stigma (Moller et al., 2021). Thus, the thematic analysis offered a contextualized exploration of the relation between participants lived experiences and the social discourse of stigma within these settings. Moreover, reflexive analysis acknowledges and values the inherent role of the researcher to analyze the data.

The analysis involved six systematic phases of a recursive, iterative line‐by‐line process, including familiarization, manually deriving data codes based on units of text pertaining to consistent issues, developing these codes into provisional themes, and refining and defining these themes (Braun & Clarke, 2006; Braun & Clarke, 2022; Byrne, 2021). The coding process was initially oriented toward a semantic approach (explicit, surface meaning) that progressed to integrating latent approaches (deeper, implicit meaning) through shared meanings when applicable. Themes reflecting stigmatizing experiences in physical healthcare settings were represented by repeated patterns of shared meaning, or a central organizing concept, generated within participant responses across the dataset (Braun & Clarke, 2022). Themes are interpreted as being both developed and distinct on their own, *as well as* in relation to other themes that contribute to an overall context and narrative. They are intended to be contextualized and multifaceted, with both data and analytical narrative, rather than merely descriptive summaries (Braun & Clarke, 2012; Braun & Clarke, 2022; Braun & Clarke, 2024; Byrne, 2021). Other preparation for analysis involved correcting minor typographical errors and defining an acronym in one written response to improve readability. Likewise, four total words from two participant responses were subtly altered or omitted to protect identifying names and hospital locations. Microsoft Excel, with the incorporation of macros, was deemed sufficient for the qualitative analysis tasks involved. The lead author, who has experience in qualitative methodology, comprising simulation exercises, team training sessions, methodology guides, and prior qualitative work, conducted this analysis. This process was completed in consultation with senior researchers with qualitative experience. The consultation was collaborative, with the goal to enhance reflexivity and solidify theme depth.

## RESULTS

Among the 81 adults surveyed, 35.8% (*N* = 29), or approximately 1 in 3 participants, reported experiencing known instances of stigma as a consequence of their BPD diagnosis or BPD symptoms while seeking care in a physical healthcare setting other than for mental health purposes.^1^ Of these 29 participants, 16 chose to disclose experiences they encountered by providing additional responses to the supplemental qualitative question. The demographic breakdown of participants is provided in Table 1.

### Thematic analysis

**FIGURE 1:**
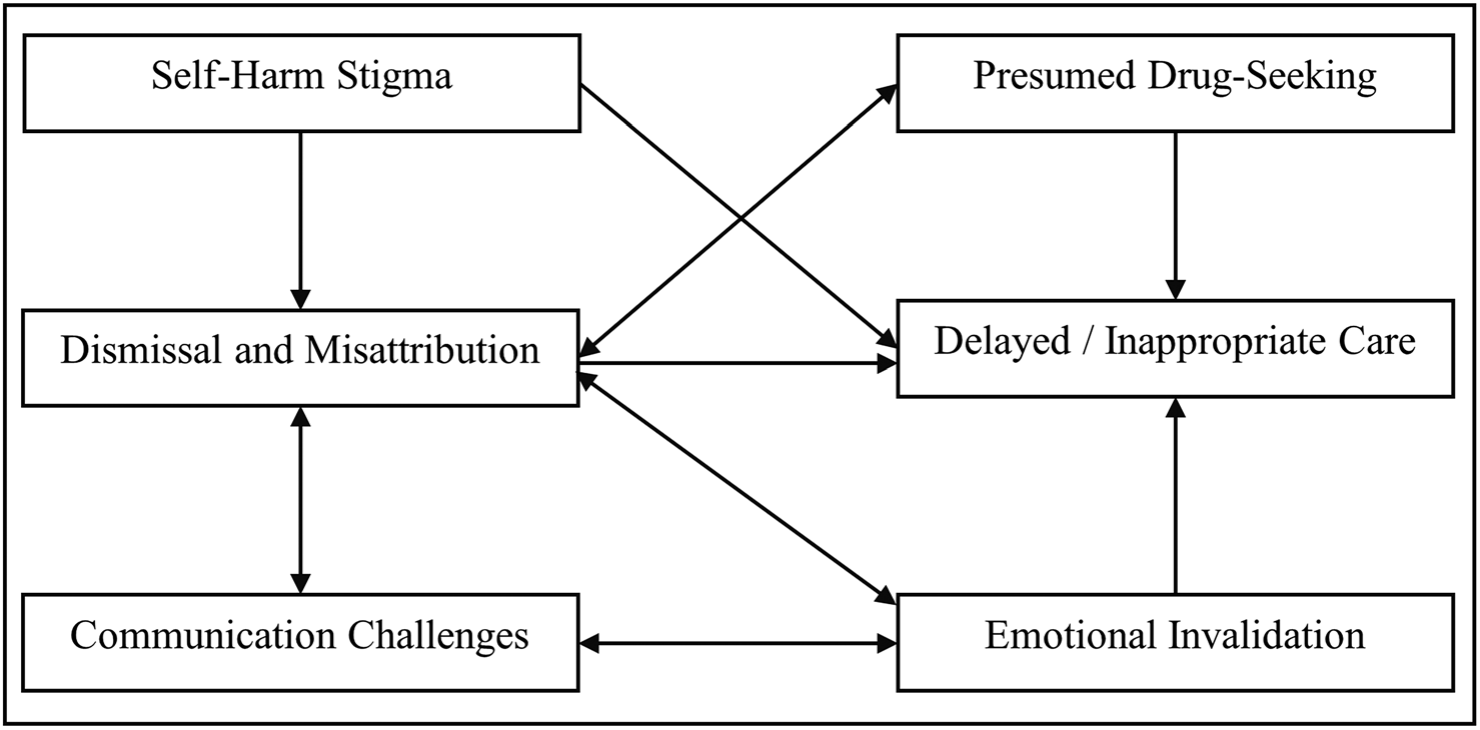
Example thematic map of the current study.

Participants recounted a variety of stigmatizing experiences during their pursuit of medical care that impacted the quality of care received, their emotional and physical well‐being, and medical outcomes. The thematic analysis yielded the following six themes: (1) Dismissal and Misattribution of Physical Symptoms, (2) Delayed or Inappropriate Medical Diagnosis and Intervention, (3) Communication and Advocacy Challenges, (4) Invalidation of Emotional Well‐Being and Distress, (5) Self‐Harm Stigma, and (6) Presumed Drug‐Seeking Behavior (see Figure 1).

#### Theme 1: dismissal and misattribution of physical symptoms

Almost all participants described experiences of their physical symptoms being dismissed or downplayed in the form of misattributions to their BPD diagnosis or BPD symptoms. One typical participant response was, “*Days after my inpatient discharge where I was dxd BPD, my PCP* [Primary care physician] *explicitly telling me, ‘it is just anxiety’ when I went to her for physical symptoms and ended up having an infection*.” Through the framing of their infection as ‘just anxiety,’ the participant evoked a sense that their health concerns were being positioned as secondary or less significant than the psychological diagnosis of BPD. Other participants were more explicit about the consistent misinterpretation of their symptoms, exemplified by sentiments such as, “*While spending 8 years seeking answers for medical problems, my BPD/cPTSD was often referenced. I was told that my allergic reactions, fainting, and joint problems were a somatic form of a BPD flare, essentially*.” Participants also talked about how BPD specifically, “*made it harder for chronic pain doctors to believe my self report of pain*.” By positioning themselves as having legitimate medical concerns that warrant consideration, participants conveyed a desire to be seen and treated as whole individuals, rather than having their physical symptoms reduced to extensions of their BPD. For participants, this meant that healthcare professionals were dismissing their physical concerns, despite the presence of them. These reports are resonant, considering the historical context of BPD in relation to stigma and discrediting their experiences of distress (see Masland et al., 2022 for a review). This recurrent pattern is related to undermined trust in healthcare providers and delayed appropriate medical treatment. The participant recounts delineated not only instances of physical misattributions but also a lack of appropriate responses to the alleged psychological symptoms, reflecting the broader issues in healthcare management for individuals with BPD.

#### Theme 2: delayed or inappropriate medical diagnosis and intervention

Participants consistently articulated the way misattributions and dismissal contributed to healthcare disparities in the form of delayed or inappropriate intervention and diagnosis, ultimately worsening their medical needs. Specifically, after medical professionals attributed their physical concerns to BPD, one participant described, “*Not ordering tests until the symptoms persisted for 3+ months*.” Other participants more explicitly also described the relation between these delays and the exacerbation of their physical and psychological well‐being. “*Attributing my real, now test confirmed physical health problems to anxiety/mood and delaying care and diagnosis, leading to losing consciousness, medical trauma, and years of damage*.” By feeling the need to emphasize the legitimacy and continuation of their physical symptoms, participants highlighted both the persistence and the delay in addressing their health concerns. These accounts demonstrated how misattributing symptoms to BPD can prevent timely and appropriate medical care, leading to consequences and complicating the individuals' overall health outcomes. Participants accentuated the need for healthcare providers to take their physical symptoms seriously and avoid defaulting to psychological explanations without comprehensive assessment.

#### Theme 3: communication and advocacy challenges

This theme encapsulates the experiences of numerous participants who encountered challenges in effectively communicating their health concerns and advocating for their needs within medical healthcare settings. Participants described difficulty with assertiveness skills when stigmatized and experiencing physical health problems (e.g., “*shutting down*” or “*screaming*”), feeling heard, and advocating for their boundaries, demonstrating the unique challenges people with BPD may experience navigating these settings. Indeed, people with BPD often have difficulty expressing intense emotions, as well as describing and labeling their experiences, which are key targets in treatments, such as dialectical behavior therapy (e.g., APA, 2022; Choudhary & Thapa, 2011; Linehan, 1993). Another participant noted how medical professionals may question third parties instead of the patient directly, stating, “*Asking others to speak for me, instead of questioning me due to heightened emotional states*.” Several other participants underlined doctors' unwillingness to attentively communicate with them regarding their concerns in relation to the insinuation that they may be fabricating symptoms due to their BPD diagnosis or expression of symptoms:

Back in 2018, I had stroke like symptoms and was losing my eye sight. Eventually, after many tests, the 1st doctor pulled my husband aside during an appointment and said to him “you sure she's not making this up? I mean she has been diagnosed with bp” my husband was extremely offended and pulled me out of there before he went off. We ended up getting 2nd and 3rd opinion. I had a tumor.

This lack of trust in the validity of their accounts, coupled with the preference for a third party to validate their claims instead of direct communication, further marginalized participants. Within the context of stigma, limited social support, and interpersonal difficulties associated with BPD (Aguirre, 2016; Lazarus et al., 2014; Lazarus & Cheavens, 2017), these accounts may speak to an increased susceptibility to adverse consequences in medical settings. Reliance on external validation to substantiate experiences is particularly concerning in the absence of these support systems. These experiences suggested a paternalistic dynamic that undermined the agency of individuals with BPD and perpetuated a sense of disempowerment within healthcare interactions.

#### Theme 4: invalidation of emotional well‐being and distress

In addition to the dismissal of physical symptoms, this theme captured the way participants' discernable emotional experiences, while experiencing physical discomfort and pain, were regularly overlooked during their healthcare encounters. Participants reported difficulty regulating their intense emotions when experiencing physical distress, with their emotions and physical symptoms further stigmatized and invalidated as a result. One participant described a hospital admission for medical purposes during which, “*I've had doctors mistreat my emotions and just label me as sensitive*.” Other times, participant emotional responses were described as, “*it's often implied I am exaggerating or lying*” about their physical pain, or “*difficulty believing me because I am acting emotional*.” These accounts revealed a recurring pattern. Participants explained that medical healthcare professionals, by stigmatizing their emotions, seemed to doubt and inadequately address their physical symptoms, which were then *also* attributed to sensitivity. Likewise, participants described the way their emotional well‐being was overlooked by providers due to stigma, resulting in heightened emotions. Whilst individuals with BPD reported high emotional reactivity, this critique situated difficulties within the fuller context of a transactional environment. These accounts are consistent with the biosocial model of BPD, which posits that recurring transactions between an invalidating environment and emotional vulnerability exacerbate and maintain symptoms (Linehan, 1993). Invalidation was framed as a core component of emotional and interpersonal difficulties, given that it signals denial or dismissal of experiences and expressions, thereby exacerbating feelings of self‐doubt and instability (Kuo et al., 2022; Linehan, 1993; Sturrock & Mellor, 2013). This invalidation of participants' emotional experiences, coupled with the challenges they faced in handling these emotions, was reported to further hinder their ability to cope, advocate, and communicate effectively. For participants, this experience was invalidating, and acknowledgment of their emotional experiences seemed to be uncommon.

#### Theme 5: self‐harm stigma

Participants expressed encounters of stigma from healthcare professionals regarding bodily self‐harm that reflected a pervasive theme within medical healthcare settings. This stigma was reported to often manifest as inadequate assessment and treatment of their injuries. One participant first lamented the experience of, “*Stigma by nurses and doctors for severe self‐harm*” indicating the initial lack of understanding from medical staff. Another participant highlighted the distressing experience of their self‐harm being left unattended to during hospital visits for medical treatment, stating, “*Not covering/treating my self‐injury marks/wounds at the hospital*.” Similarly, other participants described the tendency to not have their self‐harm injuries adequately evaluated and assessed indicating a “*Refusal to check for concussions or even just any assessment after falls/head related self harm … Talking to me differently after seeing scars*.” It is not uncommon for adults with BPD who report a history of self‐harm to describe themselves as experiencing a higher frequency of stigmatizing encounters (Veysey, 2014), such as avoidance and being thought of as “manipulative” or “attention‐seeking” (Aguirre, 2016; Linehan, 1993; Ociskova et al., 2017). These accounts suggest a significant gap in understanding and thus providing adequate medical care to individuals with BPD struggling with self‐harm. The context and description of this inadequate care indicated a risk of exacerbating their condition and exposing them to further health risks.

#### Theme 6: presumed drug‐seeking behavior

Several participants recounted instances where healthcare professionals, with the knowledge of their BPD diagnosis or BPD symptoms, prematurely anticipated that their healthcare visits were motivated by drug‐seeking behavior, unrelated to their initial medical concerns. Participants wrote of assumptions that led to judgment and skepticism regarding their intentions and medical needs. One participant, who was previously diagnosed with BPD at the same hospital, described how their physical symptoms were attributed to feeling anxious and emotional, followed by the memorable experience of, “*Assuming that I am drug seeking in the ER for unrelated issues.”* The experiences also involved:

*They said I needed to “calm down” then asked “what I came here for?” Then they asked “what do you want us to do? Give you something??” They asked me if I went to any other hospitals for “prescriptions” today. They did not seem focused* [on] *why I was there. They asked me after I already told them what happened. I turned blue when sick. I was coughing, couldn't breathe, then puked. I was woozy. I had to down to get my family. I was “blue” when she saw me*.

Here, this participant described how, even when there were signs of physical health concerns unrelated to a specific prescription, their legitimacy was still discounted. This context makes sense, given the known medical practice of suspecting that certain patients may be attempting to secure prescriptions from multiple sources (McCaffery et al., 2005; Peirce et al., 2012). Another typical participant response described that they experienced, “*Judgement on medications taken and purposes of said meds. My asking for pain meds makes me look ‘drug seeking’ and ‘impulsive’ based on BPD diagnosis*.” Although individuals with BPD may indeed have difficulties with substance use disorders (Trull et al., 2018), participant experiences maintained that medical professionals discounted the legitimacy of physical concerns to drug use and did not appropriately respond. This critique is not unreasonable. Emergency providers have shown low accuracy in identifying “drug‐seeking behavior,” and pain is one of the most common reasons overall for emergency visits (Grover et al., 2011; Weiner et al., 2013). Even when an individual with BPD presents with a comorbid substance use disorder, physical health concerns may still be and are likely to be, present (e.g., Keaney et al., 2010). Participant accounts underscored the need for improved approaches and clinical tools to evaluate and support individuals with BPD and physical concerns, both with and without substance use disorders.

## GENERAL DISCUSSION

### Current study and implications

The current qualitative study aimed to further understand stigma related to BPD in medical settings from the perspectives of participants with lived experience. The study demonstrated that the stigma associated with BPD is commonly reported across medical settings for physical healthcare and creates barriers to adequate assessment and treatment, thereby exacerbating mental and physical health outcomes. Given the elevated rates of medical concerns and increased need for healthcare among individuals with BPD (e.g., El‐Gabalawy et al., 2010), the study highlights potential implications for how these disparities may intersect with experiences of stigma in healthcare settings. Specifically, six connecting themes were produced, which attribute meaning to the way individuals with BPD navigate medical settings: (1) Dismissal and Misattribution of Physical Symptoms, (2) Delayed or Inappropriate Medical Diagnosis and Intervention, (3) Communication and Advocacy Challenges, (4) Invalidation of Emotional Well‐Being and Distress, (5) Self‐Harm Stigma, and (6) Presumed Drug‐Seeking Behavior.

The thematic examination from this study mirrors recognized stigmas associated with BPD, such as misperceptions of being attention‐seeking, exaggerating symptoms, or exhibiting manipulative intent (Aguirre, 2016; Aviram et al., 2006; Masland et al., 2022; Ociskova et al., 2017). These misattributions can lead to inadequate or inappropriate responses for both physical and mental health concerns. These themes generally shared a commonality: BPD participants reported that medical professionals seemed to assume they were distorting, exaggerating, or misleading their physical concerns, thus casting doubt on the credibility of their experiences and exacerbating their physical and emotional state. Throughout the study, participants consistently revealed that both the diagnosis of BPD, as well as the interrelated, discernable symptoms (e.g., self‐harm scars, emotional distress, interpersonal difficulties) contributed to their experiences of stigma.

The study also brings forth other implications in healthcare practice. For example, the use of medical care among patients with BPD has been described as “over‐utilized,” implying that they use medical care more than is warranted (Sansone et al., 2011). While it is true that individuals with BPD may have higher rates of medical care utilization compared to the general population, it is important to recognize that this rate may not necessarily reflect “over‐utilization,” but rather unmet or elevated physical and mental healthcare needs. In addition to lacking adequate medical care, stigma may also contribute to disengagement from treatment, difficulties with medical adherence, and eroding trust between patients and medical providers. The therapeutic relationship and rapport are significant predictors of outcomes for BPD in therapy (McMain et al., 2015). It is likely that, especially given the interpersonal difficulties with BPD, this association holds importance among physical health settings as well. The data also point to the importance of supporting individuals with BPD through stigmatizing healthcare systems. Throughout the data, participants described feelings of invalidation, emotional distress, and communication challenges. Individuals with BPD may benefit from social advocacy and the development of support and interpersonal skills.

Furthermore, the data illuminate areas where medical professionals in physical healthcare settings may benefit from enhanced education, communication, and training. Medical professionals may benefit from receiving education on the intersections of BPD and physical health outcomes (e.g., Barber et al., 2020; El‐Gabalawy et al., 2010), responding to self‐harming behaviors and the functions of these behaviors, and responding to emotional distress (Koning et al., 2017; Rees et al., 2014; van Boekel et al., 2013). Interdisciplinary team approaches to healthcare may be particularly beneficial. Medical healthcare providers should conduct clear and competent assessments of symptoms before attributing them to specific psychological causes and remain vigilant in questioning potential biases.

### Future research

Reflecting on the research process, this study prompts several considerations for conducting future research. Namely, the six themes in the study point to opportunities for future research to further examine their interconnections across different methods and contexts. In addition to this, the study reinforces the need to incorporate community‐based research models. These models, in collaboration with the impacted community, address issues relevant to the historical harms and needs of marginalized communities and offer guidance on how to do so (Bettis et al., 2023; Collins et al., 2018; NIH Principles of Community Engagement Second Edition Report, *Publication No. 11–7,782, 2011*). Research should be executed carefully to avoid reinforcing stereotypes and ensure appropriate contextualization, with the understanding that minoritized communities, compared to individuals outside those communities, may have different experiences and understandings of those experiences (Masland et al., 2022; Rodriguez‐Seijas et al., 2023). Attention is needed to ensure that research and clinical frameworks (e.g., characterizing people with BPD as “emotionally sensitive”) do not necessarily discount the lived experiences of individuals with BPD. The participant experiences in this study affirm the need to reconsider how dominant stigmatizing discourses are associated with perspectives and responses toward people with BPD, and to explore these dynamics across contextual, individual, and structural levels. There is also a need to further explore the processes and consequences of internalized, anticipated, and experienced stigmatization; limited research to date has revealed high internalization among participants with BPD (Quenneville et al., 2020). Additionally, BPD stigma has historically intersected with gendered biases, labeling BPD patients as “difficult” or “overly emotional” women (Simmons, 1992; Sulzer, 2015). Besides this, gender biases, or the tendency to dismiss or downplay women's pain and symptoms as emotional or psychosomatic, have been shown to impact healthcare delivery (Govender & Penn‐Kekana, 2008; Simmons, 1992; Sulzer, 2015; Zhang et al., 2021). Consistent with these prior studies, some participant responses may intersect with gender, as evidenced by medical professionals' responses to physical symptoms, emotions, and reliance on third‐party sources for credible information. Future research may elucidate the way demographic characteristics intersect with BPD to impact physical healthcare.

### Limitations

Limitations of the present study include the potential for restrictions inherent in the use of a standalone qualitative question, though online surveys for qualitative research are considered suitable (Braun et al., 2020). Additionally, the reliance on a limited number of questions may potentially overlook nuanced or more detailed responses that could provide deeper insights into the research topic. Although participants were comparable across characteristics, another limitation includes the potential for differences between participants who experienced stigma in medical settings and chose to disclose written responses, compared to participants who experienced stigma and chose not to disclose written responses. Nevertheless, this sufficient sample size was informed by qualitative information power, which utilizes information such as the aims of the study, the type of analysis, and the quality of the responses that generated consistent themes (Braun & Clarke, 2019; Malterud et al., 2016). Participants were also selected based on their self‐identification as having been diagnosed with BPD. While the study included several provisions, such as thorough recruitment referrals, screening items related to their diagnostic process, and measures of BPD, the absence of a formal diagnostic interview in the study remains a possible limitation. Should an individual's lifetime diagnosis not fully align with BPD, their experiences likely still reflect the lived experiences of individuals with high sub‐threshold BPD traits, who still experience significant functional difficulties (Thompson et al., 2019). There are also possible limitations on representing and discussing additional stigma experiences among individuals with BPD in medical healthcare settings, notably the relationship between ableism, health disparities, and racism (Lundberg & Chen, 2024; Priest & Williams, 2021). Lastly, the study does not assess for other factors that may influence whether or not participants endorsed experiences of stigma in medical settings, such as access to or use of healthcare. Despite these limitations, this study informs future research, and areas of improvement, and adds to the existing literature by identifying patterns of healthcare experiences and outcomes among individuals with BPD.

## REFLEXIVITY STATEMENT

As a person with lived experience of BPD, my experiences and connections to the community significantly shape my research. I am currently a graduate‐level clinical psychology researcher and clinician with a focus on BPD, deconstructing emotion [dys]regulation, suicide, self‐injury, and disability‐related issues, such as ableism and accessibility. My research is informed by my experiences and specialization in both psychology and interdisciplinary disability studies, which guide my commitment to advancing accessible care and community‐driven research models. I recognize that the effective use of *both* my lived experiences and professional knowledge is integral to my work.

Throughout the research process, I am highly self‐aware of how my lived experiences, whether they align with *or* differ from those of the participants, may influence my research questions and offer insights. My understanding of the BPD experience and disability allows me to approach research with an informed perspective and reveal issues that may not be fully appreciated or understood by those outside these communities. Several participants remarked that my questions were particularly relevant and that they were “long overdue.” It allowed them to talk about experiences and patterns of stigma related to BPD that, in their words, others might not understand to the same degree.

I have also experienced and witnessed BPD stigma across multiple settings. By asking all participants the same open‐ended question, I aimed to explore stigma‐related patterns and practical implications that impact individuals with BPD in physical healthcare settings, a concern drawing from my experiences and observations. As a result, the process was exceptionally informative to me. Additionally, I collaborated with several professionals in the field to assist with formative feedback and support, as noted in the acknowledgment section. Throughout the research process, I employed conscious reflexivity, recognizing that a researcher's intersecting identities, observations, experiences, and contributions are necessary, unavoidable, and integral to the process of reflexive thematic analysis.

## CONFLICT OF INTEREST STATEMENT

The author declares no conflict of interest.

## ETHICS STATEMENT

The study was approved by the respective Institutional Review Board.

## Data Availability

Participants granted informed consent for their responses to be utilized for research purposes, but not for data dissemination beyond the research team.

## ENDNOTE

^1^
Although BPD stigma may occur without diagnosis disclosure for reasons beyond the scope of this article, it is important to note that this study did not include data on the percentage of participants with BPD documented in their medical records. Therefore, the actual percentage of individuals with a disclosed BPD diagnosis in their medical records who reported experiencing stigma could range from 35.8% to 100%.
